# Investigations on Primary Cilia of Nthy-ori 3-1 Cells upon Cysteine Cathepsin Inhibition or Thyrotropin Stimulation

**DOI:** 10.3390/ijms24119292

**Published:** 2023-05-26

**Authors:** Alara Gaye Doğru, Maren Rehders, Klaudia Brix

**Affiliations:** School of Science, Constructor University, Campus Ring 1, D-28759 Bremen, Germany; alaraadogru@gmail.com (A.G.D.); mrehders@constructor.university (M.R.)

**Keywords:** cysteine cathepsins, E64, E64d, primary cilia, thyroid-stimulating hormone

## Abstract

In the thyroid gland, cysteine cathepsins are secreted upon thyrotropin stimulation for thyroglobulin processing, and they are present at the primary cilia of thyroid epithelial cells. Treatment with protease inhibitors resulted in the loss of cilia from rodent thyrocytes and caused redistribution of the thyroid co-regulating G protein-coupled receptor Taar1 to the endoplasmic reticulum. These findings suggest that ciliary cysteine cathepsins are important to maintain sensory and signaling properties for the proper regulation and homeostasis of thyroid follicles. Therefore, it is important to better understand how cilia structure and frequencies are maintained in human thyroid epithelial cells. Hence, we aimed to investigate the potential role of cysteine cathepsins for the maintenance of primary cilia in the normal human Nthy-ori 3-1 thyroid cell line. This was approached by determining cilia lengths and frequencies in cysteine peptidase inhibition conditions in Nthy-ori 3-1 cell cultures. Cilia lengths were shortened upon 5 h of cysteine peptidase inhibition with cell-impermeable E64. Likewise, cilia lengths and frequencies were decreased upon additional overnight treatment with the cysteine peptidase-targeting, activity-based probe DCG-04. The results suggest that cysteine cathepsin activity is required for the maintenance of the cellular protrusions not only in rodents, but also in human thyrocytes. Hence, thyrotropin stimulation was used to simulate physiological conditions that eventually lead to cathepsin-mediated thyroglobulin proteolysis, which is initiated in the thyroid follicle lumen. Immunoblotting revealed that thyrotropin stimulation conditions result in the secretion of little procathepsin L and some pro- and mature cathepsin S but no cathepsin B from the human Nthy-ori 3-1 cells. Unexpectedly, however, 24 h incubation periods with thyrotropin shortened the cilia although higher amounts of cysteine cathepsins were present in the conditioned media. These data point to the necessity of further studies to delineate which of the cysteine cathepsins plays the most prominent role in cilia shortening and/or elongation. Collectively, the results of our study provide corroboration for the hypothesis of thyroid autoregulation by local mechanisms that our group previously proposed.

## 1. Introduction

Cysteine cathepsins are an important group of proteases that regulate many physiological and pathological processes. They were initially considered to be molecules exclusively of the endo-lysosomal system; however, many studies have demonstrated that cathepsins are also found outside of endocytic compartments, such as in the nucleus and the extracellular space. In their canonical and non-canonical locations, cysteine cathepsins have been shown to be proteolytically active to different extents [1,2].

The thyroid gland, an endocrine organ composed of thyroid follicles built with a monolayer of thyroid epithelial cells (thyrocytes), is one of the organs where cysteine cathepsins play an important role in tissue homeostasis [3]. Thyroid function depends on the synthesis and proteolytic processing of the prohormone thyroglobulin (Tg). Tg synthesis, as well as Tg processing for its utilization, are stimulated by a pituitary hormone called thyroid-stimulating hormone (TSH, thyrotropin), which leads to the liberation of the thyroid hormones (TH) triiodothyronine (T_3_) and thyroxine (T_4_) from within thyroid follicles. TH are necessary not only for a functional thyroid gland but are crucial for most other cell types as well [4].

Cysteine cathepsins B, K, L, and S are involved in the proteolytic processing and degradation of Tg intracellularly within endo-lysosomes and extracellularly in the follicular lumen for initial TH liberation [5,6]. In pathological states of thyroid cells such as thyroid cancer and hypo- and hyperthyroidism, cysteine cathepsins were shown to be up- or downregulated and/or found in cellular locations different from healthy thyroid tissue cells in situ [1,7].

Primary cilia (PC), which are found in the vast majority of cell types, have significant roles in many developmental and physiological processes [8]. For instance, the primary cilium in well-polarized states of thyrocytes is linked to the maintenance of follicular homeostasis [9]. It is plausible to propose that PC extending from apical surfaces of thyrocytes into the follicular lumen sense the luminal environment by probing the molecular state of Tg [3,9,10,11]. In human diseases called ciliopathies, alterations in PC structure, frequency, or function are commonly observed [12,13]. In the thyroid gland, PC alterations typically indicate dysfunctional thyroid states or neoplastic pathologies [9,14].

A previous study from our group revealed that cysteine cathepsins B and L are localized at the cilia of Fisher Rat Thyroid (FRT) cells and that the inhibition of the cysteine cathepsins resulted in the disappearance of PC from these cells [10]. Here, we wanted to investigate the potential role of cysteine cathepsins in the maintenance of PC in the human thyroid epithelial cell line, Nthy-ori 3-1, which typically features one PC per cell. This cell line is a suitable model for studying thyrocytes, as it originates from normal thyroid tissue and responds to TSH [15]. We propose active cysteine cathepsins to maintain the PC of Nthy-ori 3-1 cells as previously described for FRT cells.

It has also been shown previously that cilia frequencies increased upon TSH stimulation in the thyroid follicular epithelia of mouse models of induced hypothyroidism (chronic high serum TSH concentrations) [14], pointing to an effect of TSH on ciliogenesis. Furthermore, TSH stimulates the activity of thyroid epithelial cells and leads to the secretion of cysteine cathepsins that mediate Tg degradation in turn [3]. Keeping these findings in mind and considering the importance of PC in maintaining a functional thyroid gland, potential alterations of PC frequencies and lengths were investigated in this study, using the normal thyroid epithelial cell line Nthy-ori 3-1 for short- and long-term TSH stimulation, mimicking regular thyroid function. To determine possible alterations in cilia lengths and frequencies upon cysteine cathepsin inhibition, cysteine peptidase activities were inhibited either using the natural inhibitors E64 and E64d, or through the activity-based probe (ABP) DCG-04 that binds irreversibly in a one-to-one fashion to the enzymes [16]. 

The indirect immunofluorescence labeling of Nthy-ori 3-1 cells and high-resolution confocal laser scanning microscopy (LSM) with z-stacking were applied to further analyze PC using CellProfiler software [17]. The expression and secretion of cathepsins B, L, and S from Nthy-ori 3-1 cells were studied by preparing whole cell lysates and TCA precipitates of proteins from conditioned cell culture media, followed by immunoblotting. Possible alterations on cilia upon TSH stimulation were investigated with indirect immunofluorescence, thereby staining for the cilia marker ADP Ribosylation Factor Like Protein 13b (Arl13b). The results suggest that cysteine cathepsin activity maintains PC in Nthy-ori 3-1 cells, though it remains elusive at this point which specific cathepsin is required. 

## 2. Results

### 2.1. DCG-04 Labels Cysteine Peptidases and Verifies Successful Cysteine Peptidase Inhibition upon E64 Treatment of Nthy-ori 3-1 Cells

**Figure 1:**
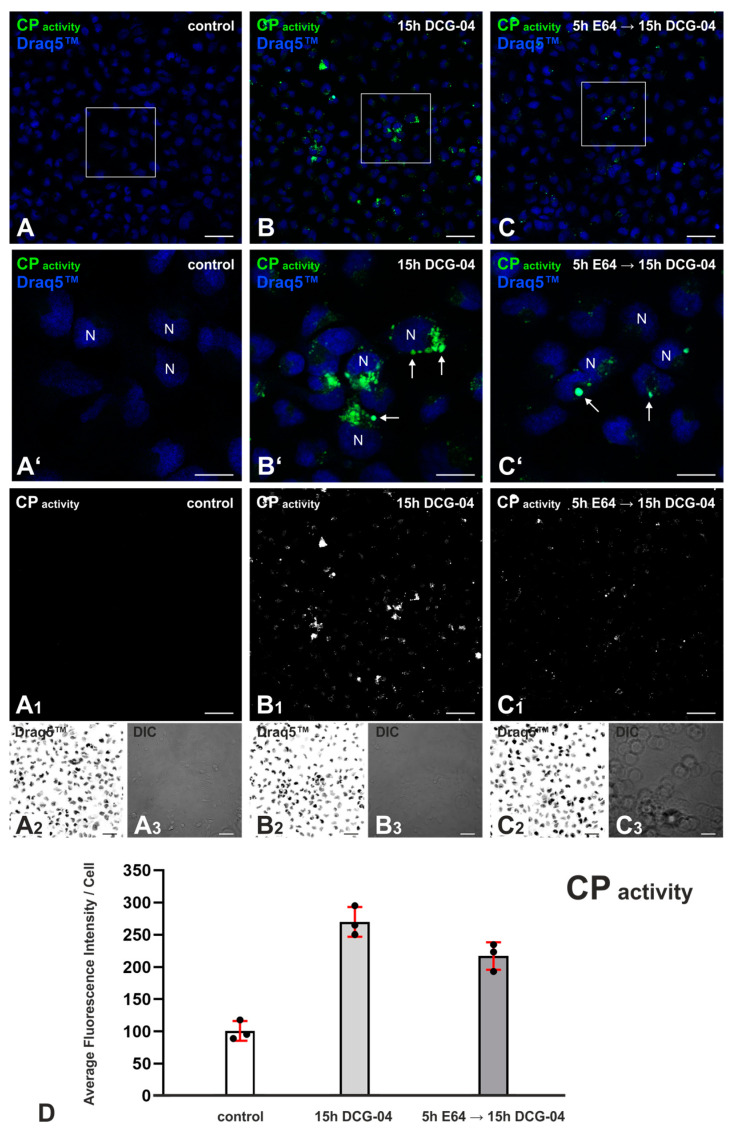
Cysteine peptidase activity upon E64 inhibition in Nthy-ori 3-1 cells. Confocal LSM micrographs of controls without either E64 or DCG-04 treatment (**A**), Nthy-ori 3-1 cells treated with a 15 h incubation period of 1 μM DCG-04 (**B**), and with a 5 h incubation period of 10 μM E64, followed by a 15 h incubation period of 1 μM DCG-04 (**C**). Merged micrographs (**A**–**C**) are shown as indicated for DCG-04, representing cysteine peptidase activity (green, CP), Draq5™-stained nuclei (N) (blue), and corresponding differential interference contrast (DIC) micrographs (**A_3_**–**C_3_**). The boxed areas in (**A**–**C**) are magnified in (**A’**–**C’**) respective to each letter. The single channel images (grayscale) for DCG-04 (**A_1_**–**C_1_**) and Draq5™ (**A_2_**–**C_2_**) are shown, the latter in inverted contrast. Arrows in (**A’**–**C’**) point to vesicular DCG-O4 signal, representative of cysteine peptidase activity. The average fluorescence intensity of the DCG-04 signal was measured using CellProfiler software. Bar charts (**D**) represent average green fluorescence intensity analyses of DCG-04 normalized to the number of cells without (middle) and upon E64 inhibition (right), revealing active cysteine peptidases. Fluorescence intensity was controlled for by not using DCG-04 or E64 (left). The data are presented as percentages of the control fluorescence intensity level. For each condition, three images were retrieved from one coverslip of the same experimental setup, and individual data points are indicated by the symbols. The sample sizes for each condition are indicated as *n* = 579 for (**A**), *n* = 613 for (**A)**, and *n* = 457 for (**C**), and they represent the total number of cells counted per condition. Nuclei were counterstained with Draq5™. Scale bars represent 50 µm in (**A**–**C**), (**A_1_**–**A_3_**), (**B_1_**–**B_3_**), (**C_1_**–**C_3_**), and 20 µm in (**A’**–**C’**).

DCG-04 is a biotin-conjugated ABP that binds to the active sites of cysteine peptidases and can, in combination with a fluorescently labeled streptavidin, be visualized via fluorescence microscopy inspection. DCG-04 irreversibly binds to cysteine peptidases in a one-to-one fashion [16]. In order to visualize active cysteine peptidases and to verify their successful inhibition upon 5 h of 10 μM E64 treatment, Nthy-ori 3-1 cells were subsequently incubated overnight with 1 μM DCG-04. Nthy-ori 3-1 cells were imaged with the LSM 980 confocal LSM (Figure 1A–C_3_), and fluorescence intensities representing cysteine peptidase activity were determined using CellProfiler-based quantification (Figure 1).

The quantitative analysis of fluorescence intensities normalized to cell numbers showed the highest values for ABP-labeled samples, as expected, and a decrease in signal intensity by approximately 20% upon pretreatment with E64 (Figure 1D). The decrease in DCG-04 intensity indicates that cell-impermeable E64 successfully inhibits the active cysteine peptidases of endo-lysosomal compartments upon internalization. The non-treated controls (Figure 1A,A’,A_1_,D) point to auto-fluorescence in the respective fluorescence channel.

### 2.2. Effects of Cysteine Peptidase Inhibition on Frequency and Length of Cilia in Nthy-ori 3-1 Cells

**Figure 2:**
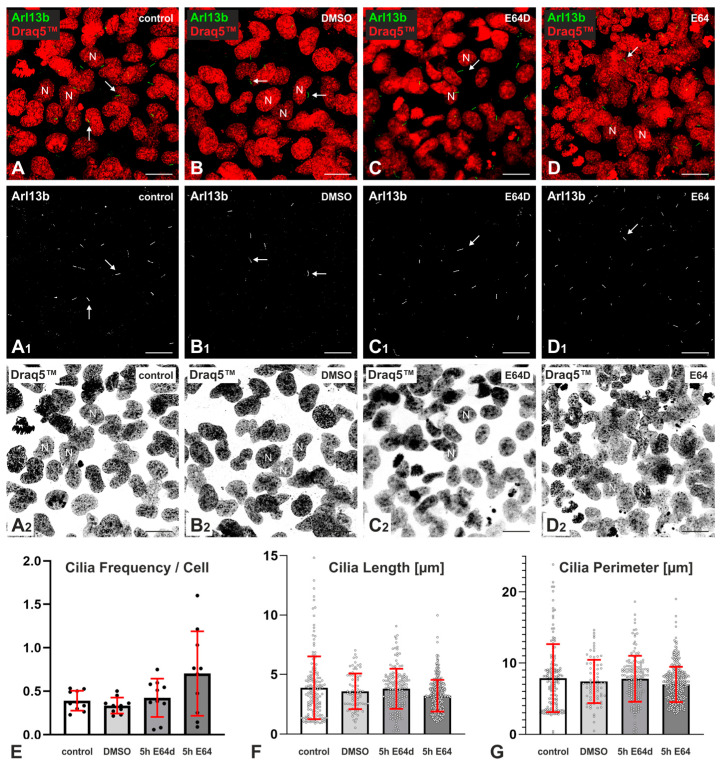
Staining and morphological analysis of cilia upon E64 and E64d inhibition in Nthy-ori 3-1 cells**.** Confocal LSM micrographs of non-treated controls (**A**), DMSO solvent controls (**B**), and Nthy-ori 3-1 cells treated for 5 h with 10 μM E64d (**C**) and with 10 μM E64 (**D**). (**A**–**D**) Merged and single channel micrographs are shown as indicated for Arl13b-stained cilia (arrows, green, white) and Draq5™-stained nuclei (N) (red, grey). (**A_1_**–**D_1_**) denote Arl13b-positive structures (cilia). (**A_2_**–**D_2_**) denote nuclei depicted in inverted contrast. Arrows in (**A**–**D**) and (**A_1_**–**D_1_**) point to cilia. The average cilia frequencies per cell (**E**), lengths (**F**), and perimeters (**G**) representative of the Arl13b signal were measured using CellProfiler software in non-treated controls, solvent controls, and E64d- and E64-treated cells as indicated. For each condition, 10 images were retrieved from two coverslips of the same experimental setup. (**E**–**G**) display the data retrieved by using bar charts, where means are represented by the height of each bar, and individual data points are indicated by symbols. The number of cells probed without inhibitor was *n* = 365; solvent control, *n* = 250; E64d inhibitor, *n* = 363; E64 inhibitor, *n* = 529. Nuclei were counterstained with Draq5™. Scale bars represent 20 µm.

Cilia of Nthy-ori 3-1 cells were immuno-stained using Arl13b-specific antibodies and visualized using confocal LSM in order to investigate potential alterations of their frequencies and lengths upon cysteine peptidase inhibition using cell-impermeable E64 and cell-permeable E64d (Figure 2). 

Analysis of cilia length and frequency using a CellProfiler pipeline (see Section 4.8) revealed that cilia frequencies were not altered by either treatment or inhibitor (Figure 2E); the lengths of cilia decreased in E64-treated Nthy-ori 3-1 cells to 3.2 ± 1.3 µm, but not in solvent-exposed controls, cultures that were treated with E64d, or non-treated controls, in which cilia were 3.6 ± 1.5 µm, 3.8 ± 1.7 µm, and 3.9 ± 2.6 µm long, respectively (Figure 2F). These data were confirmed by measuring the perimeter of cilia instead of their length as given by maximum extension (Figure 2G). Nuclear morphology did not change in both 5-hour-long E64- and E64d-treated Nthy-ori 3-1 cells (Figure 2C_2_–D_2_).

**Figure 3:**
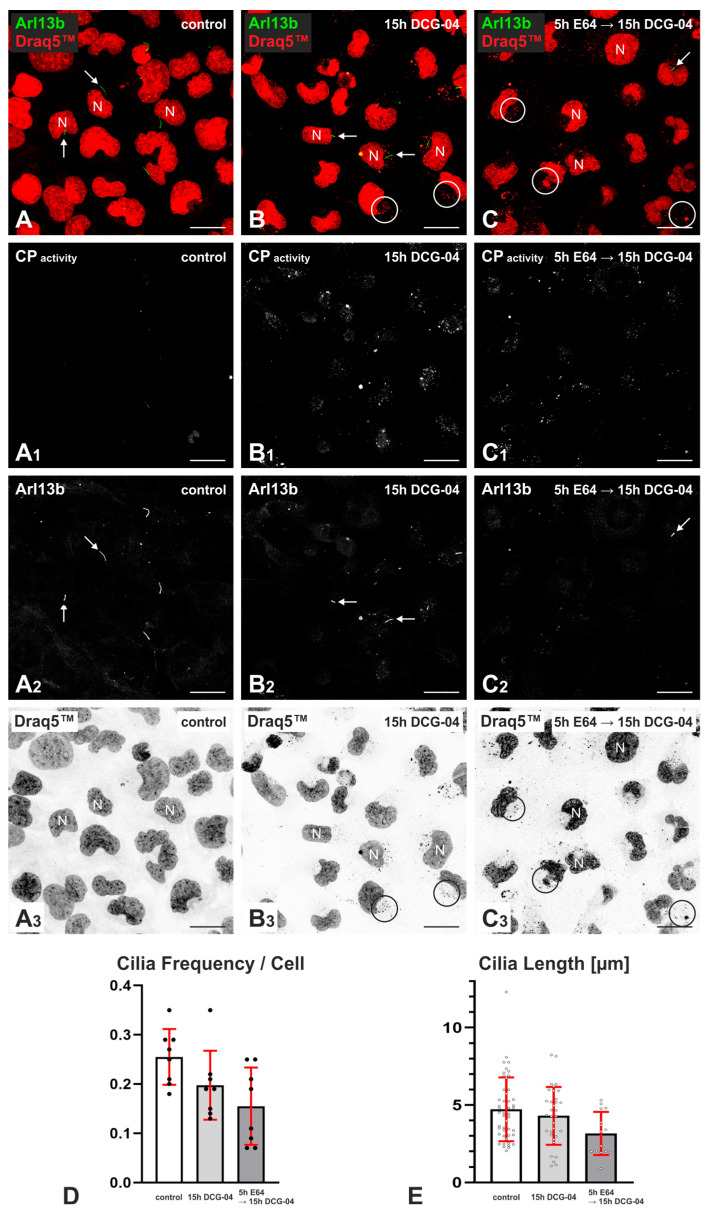
Staining and morphological analysis of cilia upon E64 inhibition in Nthy-ori 3-1 cells. Confocal LSM micrographs of controls (**A**), Nthy-ori 3-1 cells treated with a 15-h incubation of 1 μM DCG-04 (**B**), and cells with a 5-h incubation of 10 μM E64 followed by a 15-h incubation of 1 μM DCG-04 (**C**). Merged micrographs (**A**–**C**) are shown as indicated for Arl13b-stained cilia (arrows) and Draq5™-stained nuclei (N). The single channel images (grayscale) for DCG-04 representing cysteine peptidase activity (**A_1_**–**C_1_**, CP), Arl13b-stained cilia (**A_2_**–**C_2_**), and Draq5™-stained nuclei (N) (**A_3_**–**C_3_**) are shown, the latter in inverted contrast. Disruptions in nuclei (N) distributed as puncta (circles) represent non-intact nuclear architecture. Arrows in (**A**–**C**) and (**A_2_**–**C_2_**) point to cilia. The average cilia frequencies (**D**) and lengths (**E**) (representative of the Arl13b signal) were measured using CellProfiler software in non-treated controls (left), cells treated with DCG-04 (middle), and cells treated with E64 followed by DCG-04 (right), revealing changes in cilia frequency and length upon treatment. For each condition, eight images were retrieved from one coverslip of the same experimental setup. (**D**,**E**) display the data retrieved by using bar charts, where means are represented by the height of each bar, and individual data points are indicated by symbols. The sample size for each condition is indicated as *n* = 192, *n* = 170, and *n* = 95 in (**A**–**C**), respectively, and represents the total number of cells counted per condition. Nuclei were counterstained with Draq5™. Scale bars represent 20 µm.

DCG-04, the ABP that allowed the visualization of active cysteine cathepsins, also inhibits cysteine peptidases by irreversibly binding to their active sites. To investigate whether the DCG-04 had an impact on cilia frequencies and lengths, Nthy-ori 3-1 cells treated with 5-hour-long E64 followed by 15-hour-long DCG-04 incubations were compared to 15-hour-long DCG-04 incubated cells and non-treated controls (Figure 3).

The most prominent and considerably long cilia were observed to protrude from the apical surface of non-treated Nthy-ori 3-1 cells as expected (Figure 3A). Analysis of cilia length and frequency further revealed that non-treated Nthy-ori 3-1 cell cultures featured an average cilium frequency per cell of 0.26 ± 0.06, whereas the average cilia length was determined to reach 4.7 ± 2.1 µm. Nthy-ori 3-1 cells treated for 5 h with cell-impermeable E64 prior to a 15-h incubation period with DCG-04 showed a decline in average cilia frequency to 0.16 ± 0.08, whereas DCG-04 only labeled Nthy-ori 3-1 cells showed a decrease in cilia frequency with an average of 0.20 ± 0.07, both compared to non-treated Nthy-ori 3-1 control cell cultures, respectively (Figure 3D). Morphometry further revealed shortening of the PC of Nthy-ori 3-1 cells to 3.2 ± 1.4 µm by the E64 treatment prior to DCG-04 incubations, whereas cells incubated with DCG-04 only exhibited cilia with a length of 4.3 ± 1.9 µm and were thereby comparable to the non-treated controls (Figure 3E). However, when inspecting cellular morphology and visualizing nuclear DNA, both DCG-04 incubations upon or without E64 pretreatment were detrimental to nuclear architecture, as many Draq5™-positive puncta were observed in addition to irregularly shaped nuclei (Figure 3, circles). This change of nuclear morphology was most obvious in E64-pretreated cell cultures, which exhibited fewer and shorter cilia, thereby highlighting the significance of intact cilia as indicators of “healthy” and “compromised” Nthy-ori 3-1 cells. 

Collectively, these results indicate that well-extended PC protruding from the apical surface of Nthy-ori 3-1 cells are excellent indicators of intact cellular and nuclear architecture. Importantly, the length of cilia depends on active cysteine cathepsins in Nthy-ori 3-1 cell cultures. Because secretion of cysteine cathepsins from within endolysosomes was reported by us previously as a short-term response to the TSH stimulation of polarized rat thyroid epithelial cells [18], we were interested to study the impact of 4 h and 24 h TSH incubations next in an approach mimicking the physiological HPT-axis regulation of the thyroid gland.

### 2.3. Effects of TSH Stimulation on Frequency and Length of Cilia in Nthy-ori 3-1 Cells

**Figure 4:**
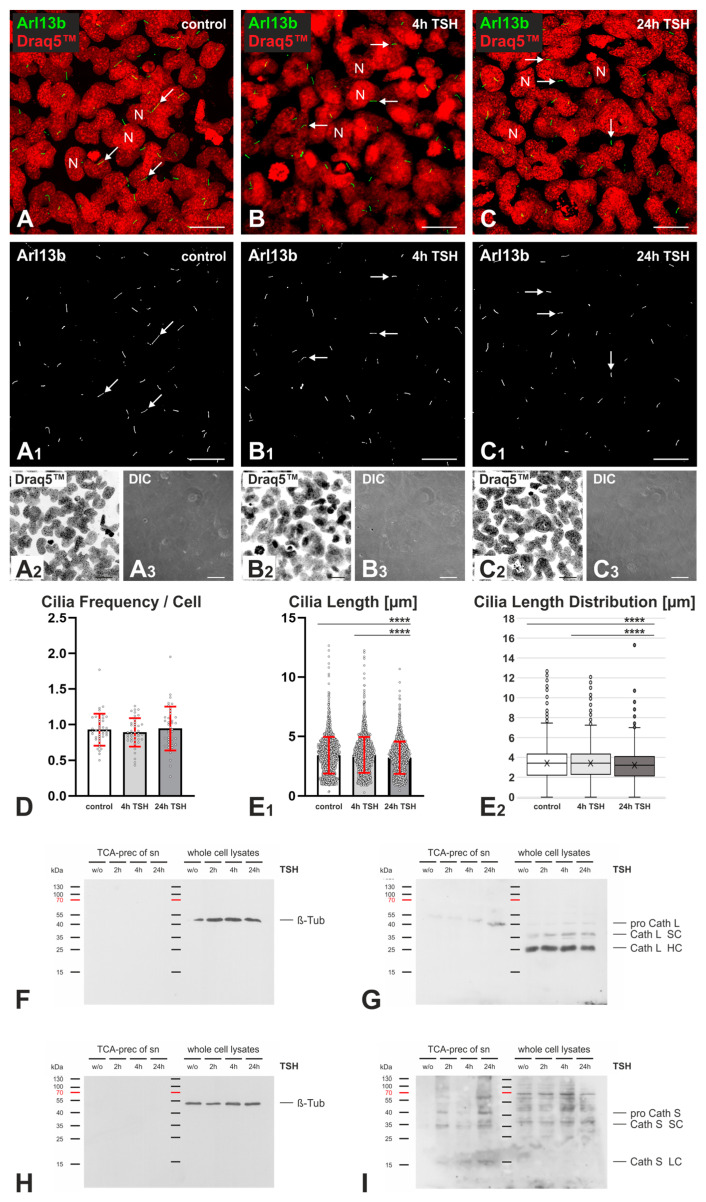
The staining and analysis of cilia and the secretion of cathepsins L and S upon TSH stimulation in Nthy-ori 3-1 cells. Confocal LSM micrographs of non-stimulated controls (**A**) and Nthy-ori 3-1 cells stimulated with TSH for 4h (**B**) and 24 h (**C**). Merged micrographs (**A**–**C**) are shown as indicated for Arl13b (green, arrows) and Draq 5™ (red), and corresponding differential interference contrast (DIC) micrographs (**A_3_**–**C_3_**) are depicted. The single channel images (grayscale) for Arl13b-stained cilia (**A_1_**–**C_1_**) and Draq5™-stained nuclei (**A_2_**–**C_2_**) are shown. Arrows in (**A**–**C**) and (**A_1_**–**C_1_**) point to cilia. For each experimental condition, a total of 40 images were obtained for each of the two replicates, each replicate comprising two slides. Nuclei were counterstained with Draq5™. The number of cells in the non-stimulated control group was *n* = 2429; cells with 4 h of TSH stimulation, *n* = 2565; cells with 24 h TSH stimulation, *n* = 2651. The number of cilia in the non-stimulated control group was *n* = 2230; in the 4 h of TSH stimulation group, *n* = 2300; in the 24 h of TSH stimulation group, *n* = 2502. The average cilia frequencies (**D**) and lengths (**E_1_**,**E_2_**) in the controls and with 4 h and 24 h of TSH stimulation were analyzed using CellProfiler software. (**D**,**E_1_**) display the data analyzed by using bar charts, where means are represented by the height of each bar, and individual data points are indicated by the symbols. (**E_2_**) The whisker plot shows the variation of cilia lengths in µm as indicated; the x denotes the median values, the boxes mark the 25th and 75th percentiles, and outliers are data points at a distance of more than 1.5 times the interquartile range. TCA-precipitated proteins from media conditioned for 48 h were collected from confluent Nthy-ori 3-1 cells, and the corresponding cell lysates upon 2 h, 4 h, and 24 h of TSH stimulation were immunoblotted with anti-β-tubulin (**F**,**H**), anti-cathepsin L (**G**), and anti-cathepsin S (**I**) antibodies. Immunoblots with anti-β-tubulin antibodies (**F**,**H**) confirmed that conditioned media were not contaminated by cell debris and that the TCA precipitates represented only the proteins secreted from the investigated cells. Cathepsin L was present in the conditioned media of Nthy-ori 3-1 cells in the proform and was present in proform (pro), single chain (SC), and heavy chain (HC) of the two-chain form in cell lysates of Nthy-ori 3-1 cells (**G**). Cathepsin S was present in the conditioned media of Nthy-ori 3-1 cells in the proform and mature forms detected at 40, 37, 35, and 17 kDa, and it was present in the 37 kDa form in cell lysates of Nthy-ori 3-1 cells (**I**). Molecular mass markers are indicated in the left margins. Scale bars represent 20 µm. Data are depicted as means ± standard deviations, **** *p* < 0.0001.

The potential alterations of cilia lengths and frequencies in well-polarized, confluent Nthy-ori 3-1 cell cultures upon TSH stimulation for 4 h and 24 h were investigated using immunofluorescence staining and CellProfiler-based densitometry of LSM images. Arl13b-positive cilia structures did not change in frequencies throughout the 4 h and 24 h TSH stimulation of Nthy-ori 3-1 cell cultures in comparison to non-treated controls (Figure 4D). The PC of non-treated controls were 3.4 ± 1.5 µm long, with mean values of 3.43 ± 1.64 µm and 3.40 ± 1.45 µm for replicates 1 and 2, respectively, which was not altered in Nthy-ori 3-1 cells with 4 h of TSH stimulation, exhibiting cilia with an average length of 3.4 ± 1.5 µm and mean values of 3.50 ± 1.59 µm and 3.39 ± 1.42 µm for replicates 1 and 2, respectively. However, the cilia length was significantly decreased to 3.2 ± 1.4 µm upon 24 h of TSH stimulation with mean values of 3.24 ± 1.39 µm and 3.19 ± 1.33 µm for replicates 1 and 2, respectively, and both with respect to non-treated or Nthy-ori 3-1 cells with 4 h of TSH stimulation (Figure 4E_1_,E_2_).

Previous data from our group show that cathepsin L was secreted from Nthy-ori 3-1 cells and present as the proform in the TCA precipitates of conditioned media, whereas the proform, single chain (SC), and heavy chain (HC) of the two-chain form of cathepsin L were detected in cell lysates of Nthy-ori 3-1 cells in steady states [7]. In this study, the cellular forms of cathepsin L were the same as previously reported, and protein amounts of intracellular forms remained unaltered upon TSH stimulation. TCA precipitates of conditioned media of Nthy-ori 3-1 cells featured the presence of procathepsin L at 42 kDa, and the amounts of TCA-precipitable procathepsin L increased in the conditioned media upon TSH stimulation for up to 24 h (Figure 4G). Similarly, procathepsin L2/V was shown previously to be secreted from Nthy-ori 3-1 cells, whereas no cathepsin B was detected in the conditioned media [7]. Cathepsin S was observed as a secretory product of human thyrocytes in situ [6], and it was found to be secreted from Nthy-ori 3-1 cell cultures in increasing amounts with longer TSH stimulation as well in this study (Figure 4I). Interestingly, cathepsin S forms with molecular masses of 40, 37, 35, and 17 kDa were detected via immunoblotting in TCA-precipitates of the conditioned media of TSH-stimulated cultures but not in the non-stimulated controls (Figure 4I). Upon TSH stimulation, both the cellular as well as the secreted cathepsin S forms increased with longer incubation times (Figure 4I), with maximum amounts in cell lysates upon 4 h and in the conditioned media upon 24 h of TSH stimulation. The data highlights TSH-regulated cathepsin S expression and secretion in Nthy-ori 3-1 cell cultures.

Thus, the results demonstrate that 24 h of TSH stimulation of human Nthy-ori 3-1 cells in vitro resulted in shortening the delicate cellular antennas protruding from the apical cell surface. The observed impact of TSH on cilia length could be correlated to procathepsin L and cathepsin S secretions from Nthy-ori 3-1 cells (see the Discussion section).

## 3. Discussion

Previous studies from our group revealed that cysteine cathepsins B, K, L, L2/V, and S are detected in the extracellular space of the thyroid follicular lumen as well as intracellularly. Keeping in mind their roles in Tg proteolysis, the enhanced extracellular activity of cysteine cathepsins may alter the luminal content and solubilize it, primarily in the pericellular space. This could be sensed by PC extending from the apical surface of the lumen-lining cells, the thyrocytes. In the present study, we tested whether an interaction of active cysteine cathepsins with PC is plausible and would result in changes to PC lengths and frequencies in Nthy-ori 3-1 cells. 

### 3.1. Inhibition of Cysteine Cathepsins Affects Cilia Frequency and Length 

Previous results from our group revealed that cysteine cathepsin inhibition resulted in the disappearance of PC from FRT cells [10]. In human diseases called ciliopathies, alterations in PC structure or frequency are commonly observed. In the human thyroid gland, such alterations have been described in thyroid disorders differing from dysfunctional thyroid states to neoplastic pathologies [9,14]. It was proven by us that cysteine cathepsins are secreted from Nthy-ori 3-1 cells in vitro [7] and from human thyrocytes in situ [6]. In analogy to previous findings with rodent thyrocytes, this could mean cilia frequency and length might depend on these cysteine cathepsin activities. Thus, the alterations of cilia frequency and length were investigated in Nthy-ori 3-1 cells upon cysteine peptidase inhibition. 

DCG-04, E64, and E64d were used for cysteine cathepsin inhibition, and their effects on cilia frequencies and lengths were studied in cultures of the Nthy-ori 3-1 cell line. The presence of active cysteine peptidases and the efficiency of cysteine peptidase inhibition by E64 and DCG-04 were verified with DCG-04 labeling (Figure 1). It is also reasonable to conclude that most of the cysteine peptidases that are inhibited were cysteine cathepsins, as other main cytosolic cysteine peptidases such as active caspases and calpains are not prominent in Nthy-ori 3-1 cells [19]. This is due to caspases being only activated upon cell death [16]. Cysteine cathepsins and legumain are the main interest in this study, as they are endolysosomal enzymes and potentially secreted from Nthy-ori 3-1 cells, unlike cytosolic caspases and calpains. Legumain is crucial for the activation of procathepsins to yield mature enzymes [20,21,22]. The inhibition of legumain might disrupt this activation process of cysteine cathepsins by impeding their ability to be rendered active. 

In analogy to previous findings, we hypothesized that active cysteine cathepsins maintain cilia in Nthy-ori 3-1 cells. The initial analysis of cilia upon E64 and E64d incubation, however, showed no decrease in cilia length upon 5-hour-long cysteine cathepsin inhibition compared to non-treated controls (Figure 2). Further analysis of cilia revealed that not only cilia lengths but also cilia frequencies were decreased when cysteine cathepsins were inhibited both with E64 for 5 h and DCG-04 for 15 h (Figure 3). DCG-04 is a derivative of E64 that irreversibly binds to active cysteine peptidases and inhibits them [16]. Therefore, it is reasonable to suggest that longer incubation times with higher concentrations of cysteine peptidase inhibitors might be needed to observe significant changes in cilia frequency and length. Longer incubation times with cysteine cathepsin inhibitors, such as E64 for 5 h and DCG-04 for 15 h, could also be the reason for changes in the nuclear morphology of Nthy-ori 3-1 cells compared to shorter incubation times with cysteine cathepsin inhibitors, such as E64 for 5 h or E64d for 5 h (Figure 2 and Figure 3). Collectively, we propose well-extended and intact PC protruding from the apical surface of Nthy-ori 3-1 cells as indicators of “healthy” Nthy-ori 3-1 cells. 

Mechanistically, E64 is likely entering cells by means of endocytic uptake similar to its biotinylated derivative DCG-04, whereas E64d acts as a hydrophobic prodrug that is converted to its inhibitory form, E64c, upon passing biological membranes. Thus, we assume that E64d may act on intracellularly present cysteine peptidases only upon its conversion to E64c, whereas both E64 and DCG-04 interacted with secreted and endocytic forms of the cysteine cathepsins. Although formal proof for this proposal is still lacking, the secretion of cysteine cathepsins was analyzed next. Cysteine cathepsins B, K, L, and S are found in thyrocytes, and they were shown to be up- and downregulated in pathological states. Cysteine cathepsins K and S were secreted from thyroid epithelial cells [23,24] and are more stable in a pericellular environment than cathepsins B and L [6,23]. In this study, cathepsin S secretion was a lot more prominent than that of cathepsin L (Figure 4). Therefore, for future studies, we suggest performing cathepsin K- and S-specific inhibition in Nthy-ori 3-1 cells to assess changes in nuclear morphology and cilia, which serve as critical indicators of deviations of thyroid homeostasis.

### 3.2. TSH Stimulation Affects Cilia Frequency and Length as Well as Cathepsin L and S Protein Amounts in Nthy-ori 3-1 Cell Cultures

Lee and colleagues demonstrated that cilia frequency was increased in the thyroid follicular epithelia of mice with induced hypothyroidism, i.e., characterized by chronically enhanced TSH concentrations in blood serum [14]. In analogy, cilia frequency and length were investigated in this study upon the TSH stimulation of Nthy-ori 3-1 cells. However, cilia frequencies did not show any change upon 4 h and 24 h of TSH stimulation, whereas cilia length was significantly decreased upon 24 h of TSH stimulation (Figure 4). The shortening of cilia could be due to TSH stimulation, which has long been known to have an effect on thyrocyte morphology [25]. TSH promotes the differentiation of thyroid follicular cells [26]. Cilia are also known to play a role in the differentiation of thyroid follicular cells [27]. Therefore, it is reasonable to conclude that there might be a direct effect of TSH on the cilia of thyrocytes.

Immunoblot analysis showed that the proform of cathepsin L was secreted to higher extents upon long-term TSH stimulation (Figure 4). This could reveal that TSH stimulates the synthesis of procathepsin L. However, this was not observed. In contrast, cathepsin S expression was enhanced upon 4 h TSH stimulation, and its secretion was reaching a maximum at the 24 h time interval (Figure 4). This is in line with the previous observation of the TSH-regulated expression of cathepsin S in the embryonic development of rats [28]. Furthermore, our group previously found the proform of cathepsin L2/V to be secreted and extracellularly matured in Nthy-ori 3-1 cells stably expressing a green fluorescent protein-tagged form [29]. Moreover, procathepsin L2 was reactive with the ABP DCG-04, suggesting that it possesses catalytic activity [29]. Therefore, the decrease of cilia length could also be due to the increase of cysteine cathepsin activity, even irrespective of the presence of mature forms, as initially hypothesized. 

Previously, we hypothesized thyroid autoregulation by local means. This proposal entailed that PC are present and extend into the thyroid follicle lumen. When the TSH receptor is activated, cytosolic calcium concentrations are raised, which trigger the secretion of cysteine cathepsins that proteolytically process the Tg to liberate TH. Tg processing eventually yields Tg fragments featuring unmasked Tg-internal sequences acting as thyropins, which inhibit cysteine cathepsins. Cathepsin inhibition triggers the disappearance of cilia. The re-synthesis of Tg completes the cycle for continuous regulation of the thyroid gland [3]. The findings of this study provide further support for this hypothesis of thyroid autoregulation through local means.

To mimic the in vivo situation of thyroid epithelial cells best, it is important to use thyropin sequences instead of the E64 inhibitor or the DCG-04 ABP. Thyropins are short peptide sequences derived from Tg that can, in principle, become unmasked upon proteolytic processing [11]. Thyropins are well-recognized for their inhibitory potential toward aspartyl and cysteine peptidases [30]. We hypothesize that using thyropins would minimize the cytotoxic effects observed in this study (Figure 3) but would provide ideal inhibitory conditions to test for the frequencies and length of PC on the apical surface of well-polarized thyrocytes. Whether changes in signaling would occur could be tested by using a cellular model with stable expression of the green fluorescent protein-tagged Taar1 G-protein coupled receptor that is trafficked to PC [31]. In this context, it is important to note that mechanistically, we propose an involvement of TH-derived metabolites such as the thyronamines [32]. Thus, it would be interesting to analyze cilia frequencies and lengths in conditions of exposure of Nthy-ori 3-1 cells to 3-iodothyronamine, which is predicted to interact with Taar1 on PC and could thereby affect their maintenance and signaling function in thyroid health and disease. In order to study pathological conditions, human KTC1 and HTh74 papillary and anaplastic thyroid carcinoma cell lines [1] are interesting cellular models for future studies.

## 4. Materials and Methods

### 4.1. Cell Culture

Normal human thyroid follicular epithelial cells (Nthy-ori 3-1 cell line) (Sigma Aldrich, Taufkirchen, Germnay, #90011609) were cultured in Roswell Park Memorial Institute medium (#RPMI-A, Capricorn Scientific, Hessen, Germany) that contains 10% fetal calf serum (Thermo Fisher Scientific, Darmstadt, Germany, #10270106, origin Brazil). Cell cultures were maintained at 37 °C in a humidified atmosphere at 5% CO_2_. For cysteine peptidase inhibition by E64 or E64d, confluently grown Nthy-ori 3-1 cells were incubated in serum-free RPMI-A for 48 h during the addition of E64 or E64d. For stimulation, confluently grown Nthy-ori 3-1 cells were incubated in serum-free RPMI-A 1640 for 48 h while being treated with 100 µU/mL TSH (Merck, Darmstadt, Germany, #869006) for up to 4 h and 24 h.

### 4.2. Protease Activity Inhibition Experiments

Cysteine peptidase activities were inhibited in confluently grown Nthy-ori 3-1 cells via incubation with 10 μM E64 (Carl Roth GmbH, Karlsruhe, Germany, #2935) or 10 μM E64d (Enzo Life Sciences, Lörrach, Germany, #BML-p1107-0001) for 5 h. As a vehicle of control for E64 or E64d, DMSO (Carl Roth GmbH, Karlsruhe, Germany, #4720.1) (0.1%, final concentration) was used.

### 4.3. Activity-Based Probes

ABPs were used to verify the successful inhibition of cysteine peptidases by E64. During 48 h of starvation of Nthy-ori 3-1 cells, cells were incubated with 10 μM E64 for 5 h followed by an overnight (approximately 15 h) incubation with 1 μM DCG-04 Green [16]. 

### 4.4. TCA Precipitation from Conditioned Media and Cell Lysate Preparation

All steps were performed on ice, and centrifugations were done at 4 °C. For cell lysate preparation, confluently grown Nthy-ori 3-1 cells were washed twice with ice-cold PBS and were harvested by using sterile cell scrapers (Sarstedt, Nümbrecht, Germany, #9095400). Cells were scraped in PBS, transferred into a pre-cooled 15 mL centrifugation tube, and collected via centrifugation for 5 min at 160× *g*. After pouring the supernatant to a fresh tube, cell pellets were resuspended in lysis buffer consisting of 0.5% Triton X-100 in PBS, pH 7.4, and a protease inhibitor mix consisting of 10 μM E64, 1 μM Pepstatin A, 0.2 µg/mL Aprotinin, and 2 mM EDTA, and the cells were homogenized for 1 min at 5000 rpm. Homogenized samples were then incubated on ice for approx. 40 min. After centrifugation at 16,100× *g* for 15 min, supernatants were transferred into a fresh reaction tube and stored at –20 °C. The Neuhoff assay was used to determine protein concentrations, and samples were normalized to equal volumes and equal amounts of proteins (20 µg, 35 µL per lane). Samples were mixed with sample buffer consisting of 1.0% sodium dodecyl sulphate (SDS), 0.124 mM Tris (pH 7.6), 50% glycerol, 0.0025 mM dithiotreitol (DTT), and 0.01% bromophenol blue.

For TCA precipitation, the conditioned media of confluently grown Nthy-ori 3-1 cells were collected and centrifuged at 160× *g* for 4 min to remove cell debris. Proteins were precipitated via incubation with ice-cold trichloroacetic acid (Carl Roth GmbH, Karlsruhe, Germany, #8789.2) at a final concentration of 10% (*v*/*v*) for 1 h. After centrifugation at 16,100× *g* for 15 min, the supernatants were discarded, and pellets were dried overnight at room temperature. The precipitated protein pellets were resuspended in SDS-PAGE sample buffer supplemented with Tris-HCl buffer 1.5 M at pH 8.8 to adjust the pH levels of TCA samples. The samples were heated for 5 min at 92 °C and stored at −20 °C until further use.

### 4.5. SDS PAGE, Semi-Dry Transfer, and Immunoblotting

All “ready-to-load” TCA-precipitate and cell lysate samples were heated at 95 °C for 5 min and loaded along with a Page Ruler Pre-stained Protein ladder (Thermo Fisher Scientific, Bremen, Germany, #26616) and separated on 12.5% resolving gels consisting of ddH_2_O, 1.5M Tris buffer (pH 8.8), acrylamide (mix37.5:1, AppliChemGmbH, Darmstadt, Germany, #A3626,1000), SDS (Omnilab, Bremen, Germany, #A7249,100), APS (Merck, Darmstadt, Germany, #1.01201.0100), TEMED (Carl Roth GmbH, Karlsruhe, Germany, #2367.3), and 3.5% stacking SDS-gels consisting of ddH_2_O, 0.5 M Tris Buffer (pH 6.0), acrylamide, SDS, APS, and TEMED in a Mini-PROTEAN 3 Electrophoresis Cell (BioRad, Hercules, CA, USA, #165-3301) at 80 V for approximately 30 min, followed by 120 V for approximately 2 h. The proteins were then transferred onto nitrocellulose membranes (Amersham Pharmacia Biotech, through Sigma Aldrich, Taufkirchen, Germany, Hybond-N+ 0.45 µm, RPN203B) via semi-dry transfer in a Trans-Blot SD Semi-Dry Electrophoretic Transfer Cell (Bio-Rad Laboratories GmbH, Munich, Germany, #170-3940) for 40 min at 25 V. The nonspecific binding of antibodies was prevented by blocking the membranes with 5% milk powder (Carl Roth GmbH, Karlsruhe, Germany, #T145.3) in PBS-T, consisting of 20 mM NaH_2_PO_4_, 0.9% NaCl, pH 6.8 supplemented with 0.3% Tween-20 (Carl Roth GmbH, Karlsruhe, Germany, #9127.2) overnight at 4 °C. Membranes were then incubated either at room temperature for 90 min or overnight at 4 °C with primary antibody dilution (diluted in PBS-T), goat anti-mouse cathepsin L (1:1000, Neuromics, Eching, Germany, #GT15049), and anti-cathepsin S antibodies (1:1000, Abcam, Cambridge, UK, #18822). After washing with PBS-T, blots were incubated with secondary antibodies, namely, rabbit anti-goat IgG (H+L) conjugated with Horseradish Peroxidase (1:2500, Southern Biotech, Eching, Germany, #6160-05) for 1 h at room temperature. Blots were washed and incubated with ECL (enhanced chemiluminescence) substrate (Thermo Scientific, Bremen, Germany, #34580) for 2–3 min at room temperature before visualization through enhanced chemiluminescence on XPosure™ films (Thermo Scientific, Bremen, Germany).

### 4.6. Indirect Immunofluorescence

In all immunofluorescence experiments, Nthy-ori 3-1 cells were fixed with 4% PFA in 200 mM HEPES at pH 7.4 for 15 min at room temperature and permeabilized with ice-cold methanol (Carl Roth GmbH, Karlsruhe, Germany, #4627.5). The slides were washed with calcium- and magnesium-free PBS (CMF-PBS), which consisted of 1.5 mM NaH_2_PO_4_, 8.1 mM Na_2_HPO_4_, 2.7 mM KCl, and 0.15 mM NaCl, for 15 min and blocked with 3% BSA (Bovine Serum Albumin, Serva Electrophoresis GmbH, Heidelberg, Germany, #11930.04) in CMF-PBS at 37 °C for 1 h. They were then washed with 0.1% BSA in CMF-PBS. For E64 and E64d inhibition experiments, rabbit anti-human/mouse/rat/dog Arl13b (1:100 or 1:200, Proteintech, Planegg, Germany, #17711-1AP) was used, followed by secondary antibody incubation, namely, goat anti-rabbit Alexa Fluor 546 F(ab’) fragments (1:200, Thermo Fisher Scientific, Bremen, Germany, #A11071). The following primary antibodies were used for TSH stimulation experiments: goat anti-mouse cathepsin B (1:40, R&D Systems, Wiesbaden, Germany, #AF965), goat anti-mouse cathepsin L (1:40, R&D systems, Wiesbaden, Germany, #AF1515), and rabbit anti-human/mouse/rat/dog Arl13b (Proteintech,1:100, Planegg, Germany, #17711-1AP). Secondary antibodies were Alexa Fluor 546 conjugated donkey anti-goat (1:200, Thermo Fisher Scientific, Bremen, Germany, #A-11056) and Alexa Fluor 488 conjugated goat anti-rabbit (1:200, Thermo Fisher Scientific, Bremen, Germany, #A-11070). All secondary antibody solutions mentioned were diluted 1:750 in 0.1% BSA in CMF-PBS. To counterstain nuclear DNA, Draq5™ (1:1000, Bio Status Limited, Shepshed Leicestershire, UK) was used at a final concentration of 5 μM. The cells grown on coverslips were rinsed in ddH2O and mounted upside-down in Mowiol (14% Mowiol 4-88, 33% glycerol, Carl Roth GmbH, Karlsruhe, Germany, #0713).

### 4.7. Image Acquisition

**Figure 5:**
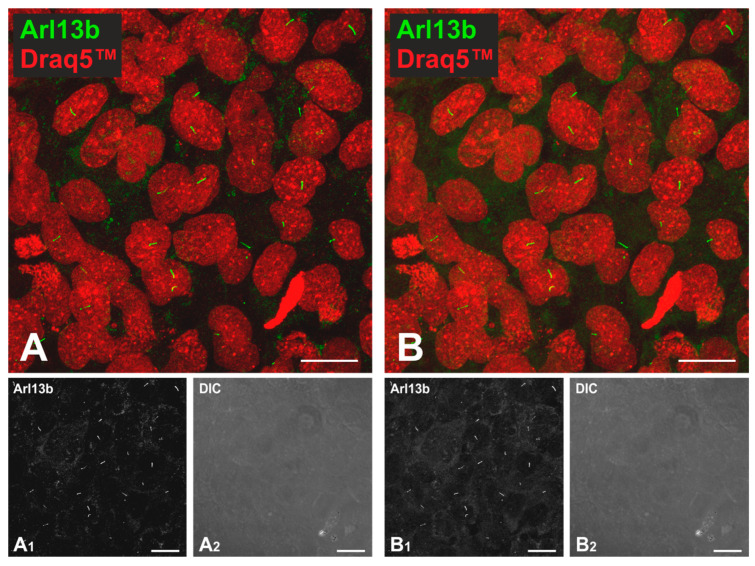
Comparison of two different Z-stack settings for best cilia visualization. Confocal LSM micrographs of the same visual field of E64-treated Nthy-ori 3-1 cells immunolabelled with rabbit anti-human Arl13b analyzed with different Z-stack settings. (**A**) Extended depth of focus image with 6 focal planes with 1.00 µm intervals. (**B**) Extended depth of focus image with 16 focal planes with 0.3 µm intervals. Single-channel of Arl13b-stained cilia (grayscale) (**A_1_**,**B_1_**), overlay of Draq5™ (red) and Arl13b (green) (**A**,**B**), and corresponding differential interference contrast micrographs (**A_2_**,**B_2_**) are depicted. Nuclei were counterstained with Draq5™ (red signals). Scale bars represent 20 µm.

All immunolabeled Nthy-ori 3-1 cells were mounted on microscope slides (Gerhard Menzel GmbH, via Carl Roth GmbH, Karlsruhe, Germany, #H868.1) and imaged with a confocal LSM equipped with Diode lasers and Diode Pumped Solid State (DPSS) lasers (LSM 980, Carl Zeiss Jena GmbH, Jena, Germany). Micrographs were obtained at resolutions of 3168 × 3168 pixels. Images were taken with the LSM 980 software, release 3.4 (Carl Zeiss Jena GmbH, Jena, Germany), and stored in CZI format and exported to TIFF format using the Zeiss Zen software, release 3.4. In order to decide on the settings of Z-stacks, two images were taken from the same region of the E64-treated Nthy-ori 3-1 cells with different interval ranges and with a different number of focal planes, namely, 6 focal planes with 1.00 µm intervals (Figure 5A) and 16 focal planes at 0.3 µm distances (Figure 5B).

The numbers of cilia and their lengths were analyzed using the CellProfiler pipeline as indicated in Section 4.8, and the results were compared to decide on suitable image acquisition settings for cilia imaging and subsequent cilia measurements. For both images, 21 cilia were detected with average cilia length of 4.0 µm for 6 focal planes (taken from the bottom of cilia to the top of cilia) and 4.0 µm for 16 focal planes (Figure 5). As both settings yielded the same results, for efficacy reasons, further Z-stacks were taken from the bottom of the cilia to the top of the cilia with a 1.00 µm interval range.

### 4.8. Image Analysis with CellProfiler

Analysis of cilia and fluorescence measurements was done using the open-source automated image analysis software CellProfiler (release 4.2.1) [17]. In order to quantify the green and red fluorescence intensities and normalize the intensities according to cell numbers, a pipeline combining “ColorToGray”, “MeasureImageIntensity”, and “IdentifyPrimaryObjects” was used. In order to quantify the cilia frequency and length, a pipeline with arrangement of different modules was used: “ColorToGray” splits the input image into respective channels: green, red, and blue, “RescaleIntensity” reduces the background intensity for red or green (cilia) and (blue) channel, “Threshold” converts the “RescaledRed” image to a binary image by applying a manual threshold value, the “IdentifyPrimaryObjects” module aims to measure the total number of nuclei and cilia, “EditObjectsManually” allows editing nuclei and cilia that were not properly selected, “MeasureObjectSizeShape” allows cilia length measurement, and “CalculateMath”, “OverlayOutlines”, “DisplayDataOnImage”, and “SaveImages, ExportToSpreadsheet” were also used. The diameters of nuclei were restricted to the range of 200–650 in pixel units, and the diameters of cilia were restricted to the range of 20–350 in pixel units. The nuclei and cilia measurements were optimized with the module “EditObjectsManually”.

### 4.9. Statistical Analyses

Fluorescence intensity measurements are shown as means ± standard deviations and as fold changes over non-treated controls. Differences among treatments and levels of significance were determined by performing Student’s *t*-test using GraphPad Prism™ (version 9.5.1; GraphPad Software Inc., San Diego, CA, USA). *p*-values below 0.05 were considered to be statistically significant. Individual data points are represented by Harvey Balls in addition to bar charts, allowing for clear identification of the values. Box-and-whisker charts were plotted using Microsoft Excel. Sample sizes and numbers of biological and technical replicates are included in the figure captions.

## 5. Conclusions

This study revealed that cathepsins L and S are secreted from Nthy-ori 3-1 cell (Figure 4). The study further investigated cilia frequency and length alterations upon cysteine peptidase inhibition with different inhibitors E64, E64d, and DCG-04, an ABP that is a derivative of E64. The results revealed that the inhibition of cysteine peptidases for longer time intervals with E64 and DCG-04 shows a decrease in cilia frequency and cilia length (Figure 3), which is accompanied by changes in nuclear morphologies, possibly indicating cytotoxicity in the treatment. In turn, this highlights the importance of well-extended cilia as indicators of healthy thyroid epithelial cell states.

**Figure 6:**
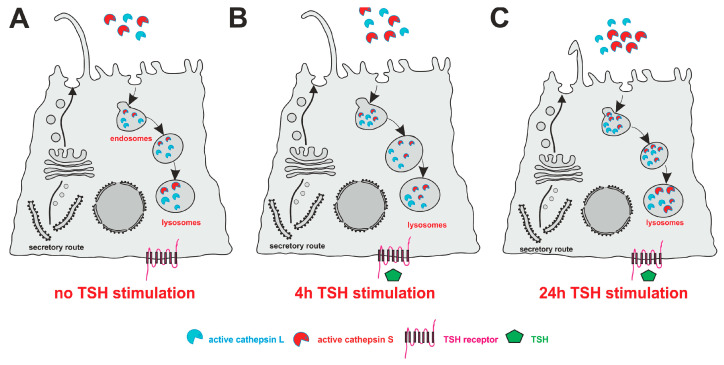
The influence of TSH stimulation on cilia length, frequency, and cathepsin L and S amounts in Nthy-ori 3-1 cells. (**A**) Resting state with basolateral TSH receptors (pink) that can be stimulated with TSH (green) in Nthy-ori 3-1 cells. (**B**) 4h of TSH stimulation in Nthy-ori 3-1 cells did not result in cilia frequency or length alterations. 4h of TSH stimulation enhanced cathepsin S expression and did not alter cellular cathepsin L amounts. (**C**) 24h of TSH stimulation of Nthy-ori 3-1 cells resulted in decreased cilia length. 24 h of TSH stimulation led to the secretion of procathepsin L, whereas no changes in cellular cathepsin L amounts were observed, and the enhancement of cathepsin S secretion and expression resulted from this long-term TSH stimulation. Note that the figure is not drawn to scale.

Another aspect of our investigation examined the impact of TSH on cilia and cathepsin L and S amounts in Nthy-ori 3-1 cells. Our results show a significant decrease in cilia length after 24 h of TSH stimulation, but no change in cilia frequencies was observed (Figure 6). The results of this study further show that cellular cathepsin L levels remained unchanged or slightly declined in Nthy-ori 3-1 cells upon TSH stimulation for up to 24 h, whereas cathepsin S expression and secretion were enhanced in both the cell lysates and the media, indicating that TSH regulates cathepsin S expression and secretion in these cells (Figure 6).

## Data Availability

All data are included in this manuscript.
